# For decades against the mainstream − From erythropoietin and hypoxia as novel treatment strategies to deep phenotyping in neuropsychiatric disorders

**DOI:** 10.1016/j.psychres.2022.114854

**Published:** 2022-09-17

**Authors:** Hannelore Ehrenreich

**Affiliations:** Clinical Neuroscience, Max Planck Institute for Multidisciplinary Sciences − City Campus, Hermann-Rein-Str.3, Göttingen 37075, Germany

**Keywords:** EPO, Functional hypoxia, Inspiratory hypoxia, Neuroprotection, Neuroplasticity, Brain doping, PGAS − phenotype-based genetic association studies, GWAS, Environment, Epigenetics, Autoantibodies

The brain has its own strategies to optimize function and plasticity in response to challenges as well as to protect nerve cells from dysfunction and destruction. Over decades, I have aimed at exploiting exactly these strategies for the treatment of neuropsychiatric patients. Using cell culture, biochemical analyses and mouse models, I studied mechanisms that are vital to nerve cell survival and functional integrity and that are potential candidates or targets for therapeutic use. Instead of focusing on one particular brain disease, I have been interested in **components of the *final common pathway*, i.e. pathophysiological mechanisms, involved in emergence and progression of diseases** like schizophrenia, multiple sclerosis, stroke, or autism. These non-disease specific mechanisms reflect a relatively uniform downstream response of the brain tissue to damage of various origins. They serve as disease-overarching targets for neuroprotective and neuroregenerative approaches, e.g. apoptosis, inflammation, compromised neuroplasticity, oxidative stress, or disturbed metabolism and homeostasis. We note that most neuropsychiatric diseases are only clinically diagnosed, based on classification systems, but etiologically and biologically heterogeneous, complex, both with respect to genetics and contributing environmental factors. They are frequent, expensive, incurable, and there is no realistic hope of a cure within the next generations. Thus, any effort to improve these conditions and to slow progression may provide a major step forward. In this part of my work, **erythropoietin (EPO) has been the most promising translational molecule in clinical neuroscience,** even though the introduction into clinical routine has not yet been as successful as would be desirable for the benefit of patients. In fact, re-combinant human (rh) EPO is an approved and safe drug for treating renal anemia since >35 years. The rhEPO market is highly lucrative, but patents expired around 2008. Industry, facing competition by biosimilar producers and fearing off-label use and emergence of new side effects, does not support research on further rhEPO indications, while funding agencies send applicants for clinical EPO projects to industry. Its name, derived from the original description in erythropoiesis, misleading for neuroscientists or reviewers, and its negative reputation as doping drug, have not helped funding research on the brain EPO system. Thus, the struggle is foredoomed.

In an **unusual ‘*human studies first*’ approach**, starting over two decades ago, I demonstrated powerful, hematopoiesis-independent effects of rhEPO on neuroprotection, neuroregeneration and cognition in humans and rodents. These highly specific and reliably reproducible effects suggested that endogenous EPO in brain serves fundamental, previously overlooked, physiological functions. Building on this work, I coined the term **‘brain EPO circle’** to explain adaptive ’brain hardware upgrade’ and enhanced performance (‘brain doping’) upon rhEPO injections or upon endogenous brain EPO induction by hypoxia. In this fundamental regulatory circle, neuronal networks, when challenged by motor-cognitive tasks, drift into transient ’functional hypoxia’, thereby triggering neuronal EPO and EPO receptor (EPOR) expression. In other words, strong motor-cognitive exercise leads to neuronal activation and functional hypoxia, provoking hypoxia-inducible factor (HIF) stabilization, followed by EPO transcription (among many other transcripts) in pyramidal neurons, which in turn grow more dendritic spines and simultaneously stimulate their neighboring cells, ready to become neurons, to differentiate within the hippocampus. In parallel, EPO reduces microglia numbers and activity, allowing the substantial amount of newly formed neurons upon brain EPO induction to be undisturbedly integrated. All this is imitated by rhEPO treatment and contributes to cognitive improvement. **Remarkably, the brain EPO circle can be entered anywhere, starting either with mild to moderate inspiratory hypoxia, with rhEPO treatment or with the aforementioned motor-cognitive challenge as inducer of functional hypoxia,** leaving plenty of possible ways and also combinations thereof for future therapeutic interventions in neuropsychiatric diseases (Ehrenreich et al., 2022, Fernandez Garcia-Agudo et al., 2021, Arinrad et al., 2021, Wilke et al., 2021, Wakhloo et al., 2020, Butt et al., 2020, Pan et al., 2019, Hassouna et al., 2016).

Whereas in this part of my work, I purposely reduce diversity and focus on common pathways to define new treatment options, in the other part, I pursue the opposite direction: In order to define etiological and biological subgroups of major neuropsychiatric phenotypes or disorders, I ignore ’disease borders’, as just artificially created by clinical classification systems. Again, the ultimate goal is to improve diagnostic and therapeutic strategies, the extreme being individualized treatments. To achieve this goal, a **better understanding of the biological basis of complex neuropsychiatric phenotypes and diseases**, such as schizophrenia, is prerequisite. This understanding is among the greatest challenges for clinical neuroscience in this century that requires novel approaches.

What we as mammals, healthy or suffering from a disease, ultimately constitute, is a highly complex ‘construct’ − and even more so as human beings. It is the synthesis of genetic make-up and personal environmental influence, ranging from e.g. birth complications to social interactions or traumatic events, mediated at least partly by epigenetic changes, even across generations. In addition, we experience a huge amount of wide-ranging outside factors like climate, nutrition, infections, population density or culture. Interestingly, also our personal repertoire of autoantibodies, directed among others against brain antigens, is shaped by the environment and may exert an epigenetic-like modulatory role. Altogether, the environmental impact on abnormal behavior and neuropsychiatric disease is enormous. **To alleviate the prevalence of mental disorders, we may even need to phenotype the environment for risk and protective factors.**

**GWAS** (genome-wide association studies, based on single nucleotide polymorphism [SNP] arrays) of complex diseases have led to genomewide significant hits (the larger the test population, the greater the number of SNPs reaching significance) of unclear relevance regarding our understanding of disease etiology. Thus, GWAS have not yet provided conclusive results to be applied for biological disease definition as prerequisite of appreciable downstream benefits for individual patients. This is mainly due to the underlying tremendous genetic heterogeneity of disease causes and the world-wide lack of deeply phenotyped samples. **Deep phenotyping** means to meticulously examine individual patients, to assess a large repertoire of functions or capacities, including e.g. sophisticated cognitive or motor performance, to explore the past and family history of these individuals, their disease course, drug and medication history, environmental influence and risk or protective factors, to just name a few examples. Only in connection with all this information, we will finally be able to make sense of genetic influence, of epigenetic modifications or of transgenerational impact.

Thus, **modification of research strategies and focused investment into phenotype-based genetic association studies (PGAS) are mandatory.** We will have to broadly perform **deep phenotyping**. It is time-consuming and labor-intense, thus not ‘sexy’, but inevitable. In fact, we have been fighting for funding of these ‘old-fashioned’ endeavors for over 20 years. In this fight, we were only ‘apparently’ running against numerous ‘multiomic competitors’, building on novel and exciting analytical technologies and ever increasing bioinformatic tools. Even though over the years, we often lost in this fight, the coin has now started to somewhat turn: What will these apparent competitors ultimately do without information on deep phenotyping? To unify GWAS and PGAS, we suggested the OTTO approach, a new strategy to extract mental disease-relevant combinations of GWAS hits from individuals. **‘OTTO’ (old Germanic = heritage) marks an individual,** characterized by a prominent phenotype and − ideally − by a family with 2nd or 3d degree relatives (i.e. only 25% or less identical genes) sharing this particular phenotype. This allows to prune for phenotype-associated risk SNPs derived from GWAS that likely contributed to the development of OTTO’s personal mental illness. The load of risk SNPs is shared by a small squad of ‘similars’, scattered under the genetically and phenotypically heterogeneous umbrella of a schizophrenia endpoint diagnosis and to a variable degree also by healthy subjects. Using the OTTO model, we extracted for *proof*-*of*-*concept* first genetic signatures of dimensional behavioral traits in schizophrenia or autism and are presently extending this approach to multiple sclerosis. Even though still in the ‘model phase’ due to a world-wide lack of sufficiently powered, deeply phenotyped replication samples, OTTO constitutes a conceptually novel strategy to delineate biological subcategories of mental diseases starting from GWAS findings and individual subjects (Janova et al., 2018, Mitjans et al., 2018, Bansal et al., 2018, Ehrenreich et al., 2018, Ehrenreich, 2017, Steixner-Kumar et al., 2021, Begemann et al., 2020).

In both main parts of my work − from EPO and hypoxia as novel treatment strategies to deep phenotyping in neuropsychiatric disorders − **the struggle will certainly continue.**
